# Impacts of residential environment on residents’ place attachment, satisfaction, WOM, and pro-environmental behavior: evidence from the Korean housing industry

**DOI:** 10.3389/fpsyg.2023.1217877

**Published:** 2023-07-28

**Authors:** Jung Young Son, Jae-Jang Yang, Sanghyuk Choi, Yong-Ki Lee

**Affiliations:** ^1^Graduate School of Business (Major in Sustainability), Sejong University, Seoul, Republic of Korea; ^2^School of Business and Graduate School of eMA, Sejong University, Seoul, Republic of Korea; ^3^School of Business and Sustainability Environment Energy Bio Institute, Sejong University, Seoul, Republic of Korea

**Keywords:** residential environment, place attachment, satisfaction, word-of-mouth, pro-environmental behavior

## Abstract

This study considers seven residential environment elements and examines their effect on residents’ place attachment (place dependence and place identity), satisfaction, word-of-mouth behavior, and pro-environmental behavior. The study also examines whether gender moderates the proposed relationships. The data were collected from 603 respondents who owned a condominium in Seoul, South Korea. We analyzed the data using structural equation modeling with SmartPLS 4. The finding shows that all seven elements of the residential environment have a significant impact on either dimension of place attachment, except for the insignificant effect of social environment on place dependence. Both dimensions of place attachment have a significant effect on satisfaction, WOM, and pro-environmental behavior except for the insignificant effect of place dependence on pro-environmental behavior. The interaction effect test of gender shows that males consider eco-friendly materials and green/recreational areas more than females. On the other hand, females are found to weigh and social environments more heavily than males. The finding shows that pro-environmental behavior is influenced by place identity (not by place dependence) and satisfaction, indicating a key role of affective response.

## Introduction

Because half of the population in Korea lives in condominiums (called “apartments” in Korea; KOSIS, 2021), the gray forest of high-rise buildings comes into mind as a first image of South Korea. The concept of residential place, in Korea, has changed from a simple space to a complex space, reflecting the characteristics of sophisticated consumers who are highly involved in the purchase process and who base their evaluation on different elements of residential environment that influence residents’ overall evaluation of the place. Residents conduct extensive information search and evaluate alternatives to find the best choice that suits their need. To attract these consumers, some residential developers in Korea have embraced environmentally friendly building material and technology to enhance their brand image and set themselves apart from the competitors by being certified as eco-friendly housing (Choi et al., 2017).

Many studies have been conducted to identify various elements (e.g., operation management, maintenance, location condition, safety management, and construction) related to residential environment (Roh and Yoon, 2022). However, very little is known about how residential environment influences residents’ satisfaction and subsequent behaviors. While some studies (Lee and Jeong, 2021) examined the effect of residential environment on place attachment, they did not use a dimensional approach for place attachment, failing to understand the mechanism, through which residential environment influences overall satisfaction and behaviors. In order to address the gap in the literature, our study proposes a framework based on the well-established hierarchy of effects model (Mehrabian and Russell, 1974; de Matos and Krielow, 2018; Lee et al., 2021). This study, based on the model, views that residents’ cognitive evaluation of the residential environment will influence affective responses (two dimensions of place attachment and satisfaction), which in turn, influence behavior. In an effort to understand the effect of residential environment on residents’ behavior, our study examines two types of consequential behaviors: their positive word-of-mouth intention (WOM) about the residential complex to other consumers and their pro-environmental behavior (PEB) which can help in the conservation of resources. In establishing the relationship, our study borrows concepts from social exchange theory (Emerson, 1976; Nunkoo and Ramkissoon, 2011) and conservation of resources theory (Hobfoll et al., 1990; Gosling and Williams, 2010). However, research on whether gender-specific differences in how However, research on whether gender-specific differences in how beliefs about the residential environment psychologically benefit person-place bonds remains unclear. The residential environment psychologically benefit person-place bonds remains unclear. Prior studies (Hwang and Ziebarth, 2006; Richardson and Mitchell, 2010; Mridha, 2020) suggest that males and females weigh residential environmental beliefs, place attachment, overall satisfaction, and consequential behavior differently. We believe that the empirical evidence will yield significant strategic implications for city developers and urban housing marketers. Furthermore, the findings of the study will have implications for policy makers and local governments seeking to incentivize developers and attract potential residents to the city area.

## Literature review

### Residential environment

Residential environment refers to factors that evaluate residential quality, which is a determinant of residential satisfaction. The residential environment is evaluated through various residential environment evaluation indicators and is used to analyze the cognitive-emotional-behavioral processes of residents (Amérigo and Aragones, 1997). For example, Craik and Zube (1976) applied the concept of Perceived Environmental Quality Index (PEQI) to evaluate the residential quality. Adriaanse (2007) measured residential satisfaction using a residential environmental satisfaction scale (RESS). Hwang (2013) suggest different elements of residential environment including housing features, security, natural environment, social network, architectural quality, pollution, transportation, commercial services, green areas, and adequate educational services, and amenities. Residential environment studies report that the quality of residential environment is strongly related to residential place attachment and satisfaction (Chen et al., 2019; Junot, 2022). Chen et al. (2019) found residents’ positive evaluation of the residential environment was a predictor of place attachment. Although residential quality itself is likely to be a major determinant of residential satisfaction, negative evaluation of the surrounding environment such as high crime rate (Mullins et al., 2001) and lack of community amenities (Fried, 1982) can cause dissatisfaction. Studies suggest that residents may use a distinct set of consideration based on the type of housing and location. For example, Mohit et al. (2010) report a positive relationship between residential environment comprising of dwelling unit features, residential unit support service, public facilities, social environment, and neighborhood facilities and residents’ satisfaction in the public housing sector in Malaysia. It is possible that residents of private housing consider a separate set of elements from those of public housing. Lee and Yeom (2011) found a positive relationship between residential environment and residents’ satisfaction using the Korea Green Building Certification Criteria (KGBCC) index. Their study that included ecological environment indicates that residents in the private housing sector may consider conservation of the environment important in their purchase decision. Lee and Choi (2013) suggest the six elements of eco-friendly housing (location condition, indoor function, brand, investment value, green space, and saving facilities) positively affect residents’ loyalty. Our study with focus on eco-friendly housing considers a comprehensive set of residential environment elements based on prior research: eco-friendly building material, management office service, dwelling unit features, public facilities, social environment, economic value, and green/recreation area. We apply the hierarchy of effects model which is widely used to explain people’s behavior in connection with cognitive and affective responses (Mehrabian and Russell, 1974; de Matos and Krielow, 2018; Lee et al., 2021).

### Place attachment

Place attachment refers to a resident’s emotional bond to a place and is comprised of two dimensions: place identity and place dependence (Kim et al., 2017; Lee et al., 2019). It facilitates to understand the integration of place beliefs, feelings, and behaviors (Canter, 1992; Jorgensen and Stedman, 2006). Place identity is a symbolic or emotional attachment of the resident to the place (Kim et al., 2017), as the resident assigns a meaning to the place, and the place becomes a part of the resident’s self-identity (Su et al., 2018) due to a sense of belongingness and the place forms a part of their self-concept (individual self-identity and social self-identity; Scannell and Gifford, 2010). According to the theory of place identity, the determination of a place identity is not solely reliant on the physical components, but also on the meaning and association established between individuals and the place (Bott et al., 2003; Lewicka, 2008). On the other hand, place dependence is an attachment formed based on function of the place that provides resources and facilities, which help residents achieve goals (Vaske et al., 2017; Lee et al., 2019). Based on Bendapudi and Berry (1997)’s (1982) study, place dependence refers to the continuation of a relationship because of limitations imposed by a specific location, wherein one party feels compelled to maintain a place-related connection due to economic, social, or psychological factors (Johnson, 1982). The degree of constraint is determined by the party’s perceived reliance on their relationship partner. Therefore, in a relationship between A and B, A’s inclination to retain the relationship based on constraints is influenced by A’s dependence on B (Dwyer et al., 1987).

## Theoretical framework

**Figure 1:**
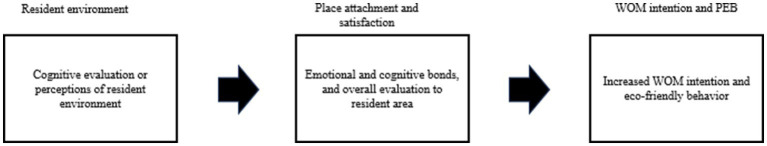
Theoretical framework.

Based on the above literature review, the study proposes a framework for testing the relationship between residential environment – place attachment – satisfaction – WOM intention and PEB. Building on the hierarchy of effects model (Smith et al., 2008; Hsiao, 2020), it considers residential environment as a cognitive attitudes or evaluation of resident. And it considers place attachment and satisfaction as an affective attitude. Finally, it considers WOM intention and PEB as a conative attitude. Building on place attachment theory (Kim et al., 2017; Lee et al., 2019), it considers why the perception of residential environment influence place identity and dependence. The framework focuses on resident’s WOM intention and PEB from and use conservation of resources theory (COR; Hobfoll et al., 1990; Gosling and Williams, 2010), which emphasizes the importance of interaction between resident and resident environment. Therefore, Figure 1 illustrates the general theoretical framework using the hierarchy of effects model. The model specifies that three components of attitude (i.e., cognitive, affective, and conative) are hierarchical (Smith et al., 2008; Hsiao, 2020). This model can be applied to understand residents’ place attachment and satisfaction. Attitude change may occur when the cognitive component is addressed first, which leads to the subsequent changes in the order of affective and behavioral components. Some critics (Barry and Howard, 1990) of the model argue that the order may change in some cases, in which the behavioral component is addressed first, followed by cognitive and affective components. For instance, a person joins his friend to run (i.e., behavioral) to realize health benefits (i.e., cognitive) and develops a positive attitude toward running (i.e., affective). Based on the hierarchy of effects model (Smith et al., 2008; Hsiao, 2020), we view that residents’ cognitive evaluation of the residential environment will influence affective responses such as a sense of attachment to the place and overall satisfaction. These affective responses are expected to influence behaviors such as WOM and PEB. Our hypotheses are discussed below.

## Development of hypotheses

### Residential environment and place attachment

We anticipate that residential environment will have a significant impact on both dimensions of place attachment (place dependence and place identity). Our rationale is as follows. If residential environment delivers expected functions or benefits to the residents, residents are likely to evaluate the environment positively and become dependent on the place. For example, a resident who enjoys the club house (one of the functions/benefits of the residential environment) to socialize with other residents may develop a sense of attachment to the place because of the function provided. We also anticipate a positive effect of residential environment on place identity. We view that residents will develop a sense of belongingness to the place when they have a positive evaluation of the residential environment (Khosravi et al., 2020).

First, whether eco-friendly materials, not endocrine disruptors, or carcinogens, are used in the residential space is a crucial factor in determining the quality of housing and has a significant impact on consumers’ housing choice and housing behavior (Lee and Choi, 2013). Therefore, whether or not eco-friendly materials are used in a product is an important factor influencing attachment (Chen et al., 2017). Since apartments are formed on a large scale, the management office service for managing apartments as multi-unit dwellings is a crucial factor constituting the quality of the living environment. Just as the services of employees affect customer attachment (Ulrich et al., 1991), the management office service in an apartment building affects residential attachment as a service to support residents (Yun and Park, 2017).

Dwelling unit features such as corridor, staircase, cleanliness of drains, street lighting, garbage collection is an important variable that constitutes the quality of the living environment of an eco-friendly apartment (Chun et al., 2008; Mohit et al., 2010; Lee and Choi, 2013; Adewale et al., 2020). Public facilities refer to the well-equipped OS/play area, parking, perimeter roads, and pedestrian walkways necessary for using the apartment complex (Mohit et al., 2010). However, in Korea, when constructing an apartment, it is mandatory to have a hall for the elderly, infant/children’s facilities, and convenience facilities for disabled people. Therefore, in Korea, the range of public facilities has been expanded, becoming an important variable that determines the residential quality of residents.

The social environment is a vital component of residential quality, such as the level of noise around or inside an apartment complex, installation and control of facilities and safety devices for accident prevention, and community relations with residents (Hidalgo and Hernandez, 2001; Mohit et al., 2010). The social environment is an open space for socializing and interacting (Binyi and Mwanza, 2014), which constitutes part of place quality and influences place identity (attachment; Isa et al., 2022). Economic value refers to the economic benefit that a resident derives from residence. Therefore, when purchasing a house, residents consider the price and ease of sale (jeonse or monthly rent) of the house (Lee and Choi, 2013). The economic value of a house is affected by the construction company and the size of the complex (Chun et al., 2008). Lastly, green/recreational area is a space that allows residents to find psychological stability against changes in the living environment, such as air pollution and temperature rise in the living space due to climate change. Therefore, residents judge the quality of housing based on whether the community they live in has enough attractive leisure spaces and green spaces, and form attachment to places (Arnberger and Eder, 2012; Li et al., 2022). Therefore, we propose that all seven elements of residential environment will influence the two dimensions of place attachment.

*H1*: Positive evaluation of the residential environment (*H1*a: eco-friendly material, *H1*b: management office service, *H1*c: dwelling unit features, *H1*d: public facilities, *H1*e: social environment, *H1*f: economic value, *H1*g: green/recreational area) influence the residents’ place dependence.

*H2*: Residential environment (*H2*a: eco-friendly material, *H2*b: management office service, *H2*c: dwelling unit features, *H2*d: public facilities, *H2*e: social environment, *H2*f: economic value, *H2*g: green/recreational area) will influence the residents’ place identity.

### Place attachment, satisfaction, WOM, and pro-environmental behavior

Place attachment is a bond to the place. Studies suggest that emotional response is an important variable that affects satisfaction, WOM, and eco-friendly behavior (Lee et al., 2019). Place attachment has a positive impact on the residents’ overall satisfaction (Fornara et al., 2019). Satisfaction is defined as a pleasurable feeling that results from the cognitive process of comparing performance against expectations (Bonaiuto and Fornara, 2004). This definition means that residents are likely to be satisfied when the performance of the residential environment exceeds their expectations. Some studies (Smith and Bolton, 2002) examine the role of emotion in satisfaction judgments and find that emotional response contribute to explaining customer satisfaction judgments, even when considering the cognitive factors that lead to satisfaction. This may be because information processing including encoding and retrieval of information is influenced by the individual’s emotional state such that individuals who are in a positive emotional state tend to rely on heuristics, mental shortcuts that allow them to make a quick decision based on the limited information (Smith and Bolton, 2002). Based on prior research on the role of emotion, we propose that place attachment (emotional bond) will have a direct influence on residents’ satisfaction judgment.

Our study anticipates that place attachment will have a significant impact on behavioral intentions (word-of-mouth and pro-environmental behavior) in addition to its impact on satisfaction judgment. Studies across disciplines support that emotion plays a key role in shaping and influencing behaviors (Kusi et al., 2021). Studies (Zhang et al., 2014) in advertising show that emotional appeals that evoke emotion are more effective at driving actions than rational appeals that require cognitive evaluations. We borrow the concepts of reciprocity, from social exchange theory (Emerson, 1976; Nunkoo and Ramkissoon, 2011) to explain our proposition that place attachment influences residents’ WOM behavior. Reciprocity is the practice of performing mutual or corresponding actions based on the other party’s actions and refers to a social norm that guides the maintenance of social relations. According to this theory, residents are likely to engage in behaviors that are beneficial to the company (e.g., WOM), when they feel positive and attached to the company (Lee et al., 2012; Chen et al., 2018). Thus, we propose that place attachment will have a positive impact on the residents’ WOM behavior.

We also anticipate place attachment to have an influence on the residents’ pro-environmental behavior (PEB) and use conservation of resources theory (COR) to explain our proposition. PEB is defined in this study as an action of residents that involves reduction of the harmful impact on the environment and contribution to the environmental conservation (Steg and Vlek, 2009; Lee et al., 2013, 2014a; Ertz et al., 2016). PEB, as described by Stern (2000), encompasses any behavior that modifies the availability of matter or energy within the environment or influences the structure and functioning of an ecosystem or biosphere in a manner that benefits rather than hampers the environment. Based on COR theory (Hobfoll et al., 1990; Gosling and Williams, 2010), resources are objects (e.g., oil), personal characteristics (e.g., leadership), and energies (e.g., time), and they are limited and scarce people try to sustain the resources that are important to them (Junot, 2022). The theory explains that if people have a close attachment to a significant other, they regard it as a value to form a social identity and are willing to preserve, maintain, love, and care for it. On the contrary, these theories explain that when the valued states are damaged, people are under stressed and act like social support so that it is not threatened. We view that residents who are attached to the place will engage in pro-environmental behavior to preserve the limited resources. There are some studies that show the positive effect of place attachment on pro-environmental behavior in the areas of festival and trading (Lee and Lee, 2020; Lee et al., 2021). For example, Lee et al. (2021) reveal that place attachment promotes visitors’ pro-environmental behavior and support for the festival. Similarly, Lee and Lee (2020) who examine the effect of place attachment in the trading area, show that place attachment (i.e., trading area attachment) has a positive influence on satisfaction, loyalty, and pro-environmental behavior.

*H3*: Place dependence influence the residents’ satisfaction (*H3*a), WOM (*H3*b), and PEB (*H3*c).

*H4*: Place identity influence the residents’ satisfaction (*H4*a), WOM (*H4*b), and PEB (*H4*c).

*H5*: Satisfaction influence the residents’ WOM (*H5*a) and PEB (*H5*b).

### The moderating role of gender in the relationship between residential environment and place attachment

Gender has been used as an important segmentation variable because males and females show different values, opinions, behaviors, and tendencies. For example, Hwang and Ziebarth (2006) show that residents’ evaluation of the residential environment differs based on gender. Some studies (e.g., Cohen et al., 2007; Richardson and Mitchell, 2010) find that men value green space more than women, while women consider neighborhood environment more important than males. Male and female residents’ satisfaction level is also found to be different. Mridha (2020) reveals that females’ housing satisfaction is higher than males. Males and females are also known to have different perceptions about the living space (Saegert and Winkel, 1980). While males view the living space as a place for work, females perceive it as a place for interaction. These previous studies suggest that males and females consider a distinct set of residential environment elements in determining place attachment. Thus, we propose that gender will play a moderating role in the relationship between residential environment and place attachment.

*H6*: The relationship between residential environment and place attachment may differ based on gender.

**Figure 2:**
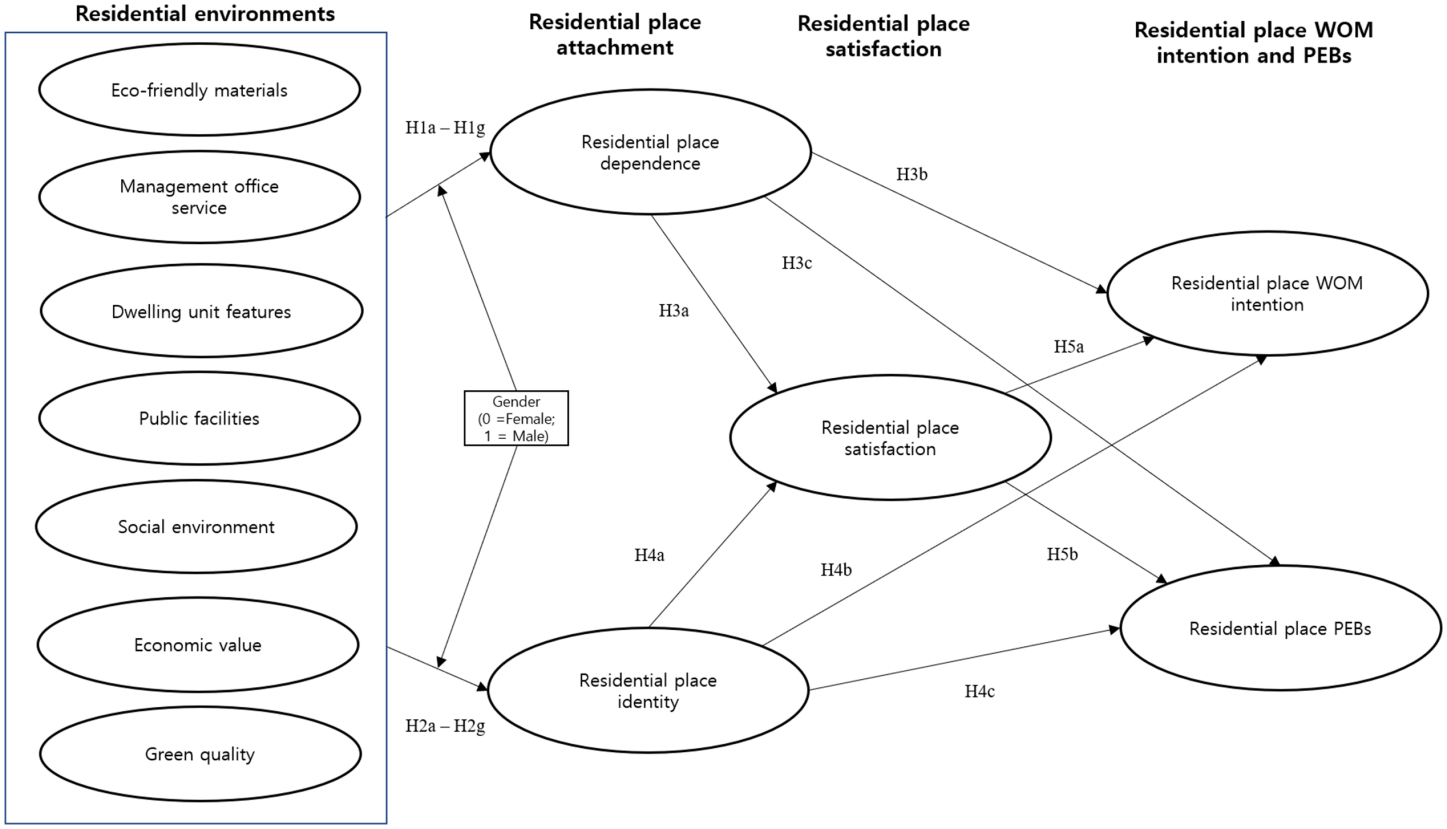
Proposed model.

Based on the hypotheses, the proposed model is shown in Figure 2.

## Methodology

### Sampling and data collection

Even though we utilized items from previous studies, they underwent modifications during a pre-test phase. We conducted a pre-test involving 10 condominium owners in order to identify any potential biases or ambiguities. The feedback obtained from the pre-test was used to modify the questionnaire accordingly. Subsequently, three experts and two academics reviewed the items to ensure their measurement appropriateness, readability, and clarity. According to their comments, some wording and sentences have been corrected.

Data were collected from condominium owners who resided in Seoul, S. Korea. We used a research company to collect data. The research company had an extensive consumer panel comprised of approximately 400,000 panel members in South Korea. The purpose of the study was explained to the participants, and they were given reassurance about the confidentiality of their information. To encourage a higher response rate, incentives were provided to participants upon successfully completing the questionnaire using an online survey company. The online survey company explained in the survey guide that the survey was conducted for academic research, that the survey results would be used only for statistical analysis, and that the panelists were to answer anonymously. The research company used simple random sampling method and reached out to 3,457 panel members, and 618 respondents completed the questionnaire. Responses with omission of essential information were excluded, leaving 603 responses qualified for final data analysis. The final sample size was 618, which exceeds the minimum requirement of 385 for a 95% confidence level and 5% sampling error.

### Measures

We used multiple items to measure all constructs. The measures were anchored by 1 (“strongly disagree” or “not at all satisfied”) and 7 (“strongly agree” or “very satisfied”). We used seven elements to measure residential environment. We borrowed the items from Mohit et al. (2010) to measure eco-friendly material (6 items), management office services (6 items), dwelling unit features (5 items), public facilities (7 items), and social environment (5 items). We adapted four items from the studies of Lundgren (2013) and Siahaan et al. (2019) to measure economic value. Green/recreational area was measured with six items (Arnberger and Eder, 2012). We borrowed items from the studies of Ramkissoon et al. (2013) and Halpenny (2006) to measure place dependence (six items) and place identity (5 items). Using the items of Lee et al. (2000), we measured satisfaction with three items. WOM intention was measured with two items based the study of Lee et al. (2014b). Finally, we used six items to measure PEB, and they were borrowed from the studies of Lee et al. (2019) and Ramkissoon et al. (2013).

### Data analysis

#### Demographic profile of the respondents

Table 1 shows the demographic profile of the respondents. The sample included a little bit more males (51.7%) than females (48.3%). About half of the respondents were in the age groups of 40s (27%), and 50s and older (26.5%). The most common occupation was professional (18.1%), followed by service industry worker (14.3%) and manufacturing employee (11.9%). About 59% of the respondents were married. Most of the respondents had a household size of 3 (35.8%) or 4 (41.8%) and obtained a college degree (73.3%). In terms of income, about 76% of the respondents earned a minimum of 5,000,000 won (approximately $3,700) a month. Most of the respondents (74%) had owned the place for less than 5 years.

**Table 1:** Demographic profile of the respondents (*n* = 603).

Category		Frequency	Percentage
Gender	Male	312	51.7
	Female	291	48.3
Age	20s	130	21.5
	30s	150	24.9
	40s	163	27
	Over 50s	160	26.5
Job	Student	47	7.8
	Manufacturing	72	11.9
	Construction	30	5
	Wholesale and retail	42	7
	Transport business	3	0.5
	Finance / Real Estate / Telecommunications	53	8.8
	Service industry	86	14.3
	Tourism (accommodation, food, and beverage)	6	1
	Professional	109	18.1
	Public official	29	4.8
	Housewife	72	11.9
	Others	54	9
Marital status	Not married	244	40.5
	Married	355	58.9
	Others	4	0.7
Number of family	1	32	5.3
	2	73	12.1
	3	216	35.8
	4	252	41.8
	5	29	4.8
	6 or more	1	0.2
Education	High school	48	8
	Two-year college	42	7
	Four-year college	442	73.3
	Graduate school	71	11.8
Monthly average income	less than 100	11	1.8
(Unit: 10 thousand won)	100–199	13	2.2
(1 dollar ≒ 1,350 won)	200–299	45	7.5
	300–499	73	12.1
	500–599	91	15.1
	600–699	158	26.2
	700–799	102	16.9
	Over 800	110	18.2
Period of residence	<3	52	8.5
(years)	3 – <5	35	65.8
	5 – <7	45	7.5
	7 – <10	56	9.3
	10 – <15	81	13.4
	15 – <20	78	12.9
	≥30	101	16.7

#### Measurement model

We performed reliability and validity tests using measurement model with SmartPLS 4.0 program (Han et al., 2022). As shown in Table 2, Cronbach’s α and composite reliability values exceeded the standard threshold of 0.7. This suggests internal consistency of the measurement model, securing morphological identity. The factor loadings and AVE values were higher than the cut-off point of 0.5, confirming convergent validity (Fornell and Larcker, 1981). As shown in Table 3, the correlation coefficients were smaller than the square root values of average variance extracted (AVE), suggesting evidence of discriminant validity. In addition, the heterotrait-monotrait (HTMT) values indicating the heterogeneity and homogeneity ratio of the correlation coefficient were lower than 0.9 (see Table 4), confirming discriminant validity. Normality was also established because the values of kurtosis (−0.477 to 0.789) and skewness (−0.750 to −0.248) were less than the absolute values of 9.0 and 2.0, respectively (Schmider et al., 2010).

**Table 2:** Measurement model.

Constructs and items	Factor loadings	Cronbach’s alpha	CR	AVE
*Residential place satisfaction*		0.910	0.944	0.848
I am satisfied with my decision to live in this area.	0.918			
Living in this area my feelings are particularly good	0.919			
I am happy to live in this area	0.925			
*Economic value*		0.854	0.895	0.630
Construction company	0.756			
Complex size	0.765			
Apartment (house) sale (jeonse or monthly rent) price	0.827			
Ease of sale (jeonse or monthly rent)	0.817			
Overall, economic value	0.803			
*Public facilities*		0.906	0.926	0.641
OS/play area	0.847			
Parking	0.720			
Hall for the elderly (new added item)	0.798			
Infant/children facilities (new added item)	0.840			
Facilities for the Disabled (new added item)	0.802			
Perimeter roads	0.753			
Pedestrian walkways	0.836			
*Management office service*		0.941	0.953	0.773
Management office staff’s knowledge of apartment management	0.870			
Kindness of the management office staff	0.896			
Courtesy of management office staff	0.887			
The management office staff respond quickly to the needs of residents	0.889			
The degree to which the management office staff is willing to help the needs of residents	0.894			
Enough staffs to serve residents	0.837			
*Green/recreational area*		0.916	0.934	0.704
The community has enough attractive recreation areas.	0.859			
My favorite recreation areas are part of the community.	0.848			
I am a regular user of the recreation areas of the community.	0.835			
The community has enough green spaces.	0.802			
I know most of the recreation areas of the community.	0.840			
I feel very safe in the recreation areas of the community.	0.849			
*Social environment*		0.877	0.911	0.672
Noise	0.735			
Accident	0.837			
Security	0.834			
Control	0.873			
Community relations	0.812			
*Eco-friendly material*		0.951	0.961	0.802
The material was made of materials that protect the environment.	0.894			
Materials will reduce the consumption of natural resources.	0.888			
The material can be recycled.	0.855			
The material is an environment-certified material.	0.910			
The material is a material that has passed an environmental audit.	0.907			
The material was made of materials for reducing (lowering) CO2 emission.	0.920			
*Residential place dependence*		0.901	0.926	0.716
I cannot think of anything better than the facilities and environment this area has to offer.	0.825			
Here you can enjoy the best environment and facilities.	0.866			
I like to live in this area more than any other area	0.852			
More than any other area, this one satisfies me more.	0.874			
This is a place where I can be comfortable.	0.811			
*Residential place identity*		0.916	0.941	0.799
This area makes me feel a strong sense of unity with me.	0.884			
This area is almost like a part of me.	0.914			
Living in this area tells me who I am. ^#^				
I am very attached to this place.	0.898			
This place means a lot to me.	0.879			
*Dwelling unit features*		0.908	0.935	0.783
Living area^#^				
Dinning space	0.854			
Bedroom space	0.873			
Toilet	0.912			
Bathroom	0.900			
*Residential place WOM intention*		0.837	0.925	0.860
I will tell the people around me about the good things about this area.	0.914			
If someone asks about choosing a residential area, they will recommend living in this area.	0.913			
*Residential place PEB (Pro-environmental behavior)*		0.881	0.913	0.677
If necessary, I will reduce my visits to my favorite places here to avoid environmental damage.	0.800			
I’m going to tell my friends not to feed the animals recklessly here.^#^				
I am signing a signature campaign to support the protection of community recreational spaces and the natural environment.	0.803			
I will try my best to know a lot about the leisure space and natural environment of the local community.	0.847			
I am willing to pay if the cost of using the leisure space and natural environment of the community is introduced.	0.814			
I will reduce my visits to my favorite places if necessary to restore the recreational spaces and natural environment of the community.	0.850			

CR, composite reliability; AVE, average variance extracted.

**Table 3:** Fornell-Larcker criterion.

Constructs	1	2	3	4	5	6	7	8	9	10	11	12
1. Eco-friendly material	**0.896**											
2. Management office service	0.498	**0.879**										
3. Dwelling unit features	0.501	0.585	**0.885**									
4. Public facilities	0.627	0.563	0.632	**0.801**								
5. Social environment	0.479	0.577	0.668	0.611	**0.819**							
6. Economic value	0.568	0.539	0.630	0.662	0.599	**0.794**						
7. Green/recreational area	0.495	0.550	0.653	0.619	0.652	0.626	**0.839**					
8. RPD	0.627	0.565	0.628	0.627	0.597	0.661	0.661	**0.846**				
9. RPI	0.542	0.583	0.598	0.553	0.588	0.606	0.700	0.765	**0.894**			
10. RPWOM	0.449	0.582	0.659	0.558	0.641	0.651	0.725	0.752	0.763	**0.921**		
11. RPPEB	0.453	0.561	0.609	0.544	0.630	0.594	0.736	0.702	0.730	0.759	**0.927**	
12. RPSAT	0.466	0.556	0.498	0.514	0.497	0.492	0.554	0.490	0.541	0.534	0.513	**0.823**
Mean	4.18	4.66	4.95	4.69	4.61	4.79	4.93	4.43	4.68	4.94	4.86	4.78
SD	1.34	1.21	1.14	1.25	1.20	1.21	1.19	1.21	1.25	1.22	1.22	1.07

Bold: The square root of the variance shared between the constructs and their measures (AVE). All correlations coefficients are significant at the level of *p* = 0.001. RPD, residential place dependence; RPI, residential place identity; RPSAT, residential place satisfaction; RPWOM, residential place WOM; RPPEB, residential place pro environmental behavior.

#### Common method bias assessment

Following Kang et al. (2021)’s method, we used procedural and statistical approaches to check for common method bias. We used three procedural methods (Podsakoff et al., 2003, 2012). The first one was involved with using a pre-test, based on which we modified words, phrases, and sentences to reduce ambiguity and enhance clarity. The second approach was to inform respondents of the study purpose. The third approach was to change the order of the independent variables, mediators, and dependent variables so that the respondents could not guess the relationship among the variables. As for the statistical approach, we checked the variance inflation factor (VIF) values against the 3.3 threshold (Kock, 2015; Wang et al., 2022). Because the VIF values (1.825–2.997) were below 3.3, common method bias was not a threat to our study.

**Table 4:** Heterotrait-monotrait ratio (HTMT).

Constructs	1	2	3	4	5	6	7	8	9	10	11	12
1. Eco-friendly material												
2. Management office service	0.525											
3. Dwelling unit features	0.539	0.632										
4. Public facilities	0.676	0.607	0.695									
5. Social environment	0.520	0.626	0.744	0.673								
6. Economic value	0.625	0.595	0.711	0.745	0.681							
7. Green/recreational area	0.530	0.591	0.715	0.672	0.723	0.701						
8. RPD	0.681	0.612	0.693	0.690	0.663	0.746	0.724					
9. RPI	0.580	0.627	0.655	0.600	0.650	0.675	0.761	0.840				
10. RPSAT	0.482	0.628	0.724	0.606	0.713	0.729	0.791	0.827	0.835			
11. RPWOM	0.508	0.632	0.698	0.618	0.730	0.698	0.837	0.807	0.833	0.869		
12. RPPEB	0.508	0.609	0.556	0.571	0.560	0.564	0.615	0.551	0.600	0.594	0.596	

RPD, residential place dependence; RPI, residential place identity; RPSAT, residential place satisfaction; RPWOM, residential place WOM; RPPEB, residential place pro environmental behavior.

#### Structural model assessment

We evaluated the fit of the model using SmartPLS 4.0 program (Hair et al., 2019; Hur and Lee, 2021; see Table 5). The finding that all VIF values were lower than 3.3 indicates no problem of multicollinearity between the constructs. The predictive fit of the model was considered appropriate because the values associated with the Stone-Geisser’s test (*Q*^2^) were higher than 0. In addition, the *R*^2^ values were greater than 0.10 (Falk and Miller, 1992), confirming the explanatory power of the model. Finally, the standardized root mean squared residual values were less than 0.1, indicating an appropriate model fit (Hu and Bentler, 1998).

**Table 5:** Structural estimates (PLS).

		Model 1				Model 2			
	Paths	Estimate	*t*	*p*		Estimate	*t*	*p*	
H1-1	Eco-friendly material → RPD	0.246	5.811	0.000	*p* < 0.001	0.225	3.829	0.000	*p* < 0.001
H1-2	Management office service → RPD	0.084	1.853	0.064	n.s	0.122	1.840	0.066	n.s
H1-3	Dwelling unit features → RPD	0.114	2.325	0.020	*p* < 0.05	0.109	1.636	0.102	n.s
H1-4	Public facilities → RPD	0.048	0.859	0.390	n.s	−0.095	1.201	0.230	n.s
H1-5	Social environment → RPD	0.061	1.361	0.173	n.s	0.091	1.395	0.163	n.s
H1-6	Economic value → RPD	0.192	4.085	0.000	*p* < 0.001	0.206	3.038	0.002	*p* < 0.01
H1-7	Green/recreational area → RPD	0.229	4.043	0.000	*p* < 0.001	0.324	4.723	0.000	*p* < 0.001
H2-1	Eco-friendly material → RPI	0.155	3.445	0.001	*p* < 0.01	0.077	1.340	0.180	n.s
H2-2	Management office service → RPI	0.170	3.802	0.000	*p* < 0.001	0.141	2.372	0.018	*p* < 0.05
H2-3	Dwelling unit features → RPI	0.073	1.478	0.139	n.s	0.073	1.069	0.285	n.s
H2-4	Public facilities → RPI	−0.055	0.997	0.319	n.s	−0.122	1.895	0.058	n.s
H2-5	Social environment → RPI	0.067	1.449	0.147	n.s	0.123	2.035	0.042	*p* < 0.05
H2-6	Economic value → RPI	0.133	2.692	0.007	*p* < 0.01	0.106	1.596	0.110	n.s
H2-7	Green/recreational area → RPI	0.390	6.692	0.000	*p* < 0.001	0.543	8.483	0.000	*p* < 0.001
		0.407	8.608	0.000	*p* < 0.001	0.407	8.605	0.000	*p* < 0.001
H3-1	RPD → RPSAT	0.180	3.453	0.001	*p* < 0.01	0.180	3.453	0.001	*p* < 0.01
H3-2	RPD → RPWOM	0.078	1.171	0.241	n.s	0.078	1.172	0.241	
H3-3	RPD → RPPEB	0.451	9.337	0.000	*p* < 0.001	0.451	9.337	0.000	*p* < 0.001
H4-1	RPI → RPSAT	0.278	4.078	0.000	*p* < 0.001	0.278	4.078	0.000	*p* < 0.001
H4-2	RPI → RPWOM	0.282	3.672	0.000	*p* < 0.001	0.282	3.672	0.000	*p* < 0.001
H4-3	RPI → RPPEB	0.412	6.950	0.000	*p* < 0.001	0.412	6.950	0.000	*p* < 0.001
H5-1	RPSAT → RPWOM	0.261	3.365	0.001	*p* < 0.01	0.261	3.365	0.001	*p* < 0.01
H5-2	RPSAT → RPPEB	0.246	5.811	0.000	*p* < 0.001	0.029	0.543	0.587	n.s
						−0.052	0.948	0.343	n.s
	Gender * Eco-friendly material → RPD					0.336	3.260	0.001	*p* < 0.01
	Gender * Eco-friendly material → RPI					0.177	1.727	0.084	n.s
	Gender * Management office service → RPD					−0.022	0.237	0.813	n.s
	Gender * Management office service → RPI					0.060	0.628	0.530	n.s
	Gender * Dwelling unit features → RPD					0.001	0.008	0.994	n.s
	Gender * Dwelling unit features → RPI					0.051	0.549	0.583	n.s
	Gender * Public facilities → RPD					−0.088	1.013	0.311	n.s
	Gender * Public facilities → RPI					−0.164	1.835	0.067	n.s
	Gender * Social environment → RPD					−0.248	2.530	0.011	*p* < 0.05
	Gender * Social environment → RPI					−0.417	4.064	0.000	*p* < 0.001
	Gender * Economic value → RPD					−0.067	0.786	0.432	n.s
	Gender * Economic value → RPI					0.072	0.877	0.380	n.s
	Gender * Green/recreational area → RPD					0.066	0.756	0.450	n.s
	Gender * Green/recreational area → RPI					0.209	2.223	0.026	*p* < 0.05
	Gender → RPB					0.225	3.829	0.000	*p* < 0.001
	Gender → RPI					0.122	1.840	0.066	n.s
		*R*^2^	*Q*^2^			*R*^2^	*Q*^2^		
	RPD	0.618	0.600			0.638	0.531		
	RPI	0.589	0.570			0.622	0.481		
	RPSAT	0.650	0.572			0.650	0.595		
	RPWOM	0.642	0.546			0.642	0.557		
	RPPEB	0.330	0.366			0.330	0.372		

^***^p < 0.001, ^**^p < 0.01, ^*^p < 0.05; n.s., not significant; RPD, residential place dependence; RPI, residential place identity; RPSAT, residential place satisfaction; RPWOM, residential place WOM; RPPEB, residential place pro environmental behavior; Gender (0 = Female, 1 = Male).

### Hypotheses testing

#### Main effect test

Hypothesis 1 states that seven elements of the residential environment will have a positive impact on place dependance. As shown in Table 5 (see Model 1), eco-friendly material (*β* = 0.246, *p* < 0.001), dwelling unit features (*β* = 0.114, *p* < 0.05), economic value (*β* = 0.192, *p* < 0.001), and green/recreational area (*β* = 0.229, *p* < 0.001) have a has a significant impact on place dependence. Therefore, *H1*a, *H1*c, *H1*f, and *H1*g are supported. Meanwhile, management office service (*β* = 0.084, n.s.), public facilities (*β* = 0.048, n.s.), social environment (*β* = 0.061, n.s.) did not have a significant impact on place dependence. Hence, *H1*b, *H1*d, and *H1*e are not supported.

Hypothesis 2 addresses that seven elements of residential environment will have a positive impact on place identity. Eco-friendly material (*β* = 0.155, *p* < 0.01), management office service (*β* = 0.170, *p* < 0.001), economic value (*β* = 0.133, *p* < 0.01), and green/recreational area (*β* = 0.390, *p* < 0.001) have a has a significant impact on place identity. Therefore, *H2*a, *H2*b, *H2*f, and *H2*g are supported. However, dwelling unit features (*β* = 0.073, n.s.), public facilities (*β* = −0.055, n.s.), and social environment (*β* = 0.067, n.s.) did not have a significant impact on place dependence. Hence, *H2*c, *H2*d, and *H1*e are not supported.

Hypotheses 3 posits that place dependence will influence residents’ satisfaction, WOM intention, and PEB. Place dependence has a significant impact on satisfaction (*β* = 0.407, *p* < 0.001), WOM intention (*β* = 0.180, *p* < 0.01). However, place dependence did not have a significant impact on PEB (*β* = 0.078, n.s.). Therefore, *H3*a and *H3*b are supported, but not supporting *H3*-3.

Hypotheses 4 presents that place identity will influence residents’ satisfaction, WOM intention, and PEB. Place identity has a significant impact on residents’ satisfaction (*β* = 0.451, *p* < 0.001), WOM intention (*β* = 0.278, *p* < 0.001), and PEB (*β* = 0.282, *p* < 0.001). Therefore, *H4*a, *H4*b, and *H4*c are supported. Lastly, Hypothesis 5 proposes that residents’ satisfaction will influence WOM intention and PEB. The study finds that satisfaction has a positive impact on WOM intention (*β* = 0.412, *p* < 0.001) and PEB (*β* = 0.261, *p* < 0.001). Hence, *H5*a and *H5*b were supported.

#### Interaction effect analysis of gender for RQ

The study, using the SmartPLS 4.0 program, examined the interaction effect of gender to identify the moderating role of gender in the structural relationship between residential environment and place attachment. As shown in Table 5 (see Model 2), the study finds that the effect of public facilities (*β* = 0.336, *p* < 0.001) on place dependence, and eco-friendly material (*β* = 0.209, *p* < 0.05) on place identity were stronger for males than females. Meanwhile, the effect of green/recreational area on place dependence (*p* < 0.05) and place identity (*p* < 0.01) is also found to be greater for females than males. Therefore, *H6* was partially supported.

## Discussion

This study using a comprehensive set of residential environment elements, finds that each element of the residential environment influences the two dimensions of place attachment differently for males and female residents. Social environment is found to have no impact on place dependence and identity for residents. The study finds that eco-friendly material, dwelling unit features, economic value, and green/recreational area are the drivers of place dependence. In other words, residents as a whole value these four elements more than other elements in shaping their dependence on the place.

Meanwhile, eco-friendly material, management office service, and economic value are the drivers of place identity. The findings indicate that residents as a whole value these three elements more than other elements in shaping their identity on the place.

Our study shows that males and females show a difference in assessing a couple of residential environment elements. While males consider public facilities concerned with parking, children-related facility, road, and sidewalk important in shaping their dependence on the place, females do not. Females consider green/recreational area important in affecting their dependence on the place, while males do not. It is possible that females utilize the recreational space more often than males, and, thus, place more weights on green/recreational area.

The study finds that green/recreational area and eco-friendly material are the driving forces behind residents’ place identity for females. This means that female residents view these elements in forming their self-identity as these elements have a special meaning to them. Green/recreational area is shown to have a greater impact on females than males in forming place identity. This result is consistent with the impact of green/recreational area on place dependence. Males consider eco-friendly material more in forming place identity than females.

This study finds that place dependence and identity have differently influence satisfaction and WOM intention. This means that different dimensions of place attachment may drive resident’s overall feelings and WOM behavior. Our interpretation is that those residents value functions and symbolic meanings of the place in evaluating places and in determining WOM behavior. The study finds that the effect of place identity on pro-environmental behavior is significant. While the effect of place dependence on pro-environmental behavior is not significant, the effect of place identity on pro-environmental behavior is not significant is significant. Place identity is an important predictor of pro-environmental behavior.

## Implications

### Theoretical implications

This research makes some theoretical contributions to the literature by drawing from concepts from environmental psychology and marketing (Mehrabian and Russell, 1974; de Matos and Krielow, 2018; Lee et al., 2021), included elements related to environmentalism (i.e., green/recreational area and eco-friendly material), house features, social aspect, public facilities around the place, value, and service. By embracing various elements, the study reveals relative impacts of the residential environment elements on place attachment. The residential environmental elements that are most important in influencing two dimensions of place attachment are eco-friendly material and green/recreational area. Green/recreational area is considered more important to females than males in determining place attachment. On the other hand, eco-friendly material is more important to males than females in influencing place identity. Another crucial element is economic value which is based on residents’ cognitive evaluation of the offerings, and is found to be a crucial element that influences place dependence, not place identity.

Another contribution of the study is related to the integration of cognitive, affective, and conative components of attitude. This study, based on the model of hierarchy of effects (Smith et al., 2008; Hsiao, 2020), identified how cognitive evaluation of the residential environment elements influences affective responses (place attachment and satisfaction), which in turn, influence behavior (WOM intention and PEBs). Thus, the findings support the model of hierarchical effects and suggest that future studies may want to consider cognitive and affective responses in studying residents’ behaviors. Using the reciprocity principle of the social exchange theory (Emerson, 1976; Nunkoo and Ramkissoon, 2011), this study examined the role of affective responses in influencing WOM behavior. The significant result suggests that the reciprocity principle is applicable to the residential studies. Based on place attachment theory (Kim et al., 2017; Lee et al., 2019). And conservation of resources theory (Hobfoll et al., 1990; Gosling and Williams, 2010), our study anticipated that residents who are affectively attached to the place will be involved in pro-environmental behavior. The significant finding suggests that conservation of resources theory is helpful for explaining residents’ pro-environmental behavior. The finding that place identity (not place dependence) and satisfaction are important predictors of pro-environmental behavior suggests that positive affective responses are a determinant of pro-environmental behavior.

Finally, this study makes a theoretical contribution by showing differences between males and females in their assessment of the residential environment elements and place attachment. The differences are found in their assessment of public facilities, eco-friendly material, and green/recreational area. The gender-based differences suggest that males and females have a different attitude toward eco-friendly housing and consider different elements in evaluating the place. Future research may be needed to understand the underlying causes of the differences.

### Practical implications

This study offers several practical implications. First, the study finding related to the importance of eco-friendly material and green/recreational area, suggests that developers should use appropriate material and design to label the housing as eco-friendly. In creating a marketing communication material, developers may want to emphasize the fact they address environmentalism by using eco-friendly material and offering green/recreational spaces. Residents are also found to consider economic value a crucial factor. Developers may want to emphasize many different economic benefits associated with eco-friendly housing in their promotional material.

The differences between males and females have some practical implications. The finding shows that males value eco-friendly material and public facilities more than females. On the other hand, females consider green/recreational area environment more than males. The finding suggests that developers and marketers focus on green/recreational area in their communication to females as it impacts their place dependence and identity and thereafter their WOM intention. For example, a message to female buyers may want to emphasize safety and security of the community and quiet environment. If the main decision-maker is a male, the message may be modified to focus on building material (e.g., eco-friendly material) and public facilities (e.g., abundant and convenient parking spaces). The study finding suggests that developers and marketers tailor their offerings and communication based on gender and consider the diverse needs of males and females in designing and building residential complexes.

One of the interesting findings is related to the effect of gender on pro-environmental behavior. While pro-environmental behavior is driven by overall satisfaction and place identity. Place dependence is found to have no impact on pro-environmental behavior. As discussed before, place dependence is related to function of the place. Satisfaction and place identity are concerned with pleasurable feelings and emotional attachment. Emotional response is critical in transforming residents’ behavior. This finding suggests that developers should make efforts to establish an emotional tie with the residents. Residents’ pro-environmental behavior and WOM are especially important to local governments in rural areas who are faced with declining population and rising debts. Our study finding suggests that pro-environmental behavior can be shaped, and affective responses are critical for influencing the behavior. Given that eco-friendly material and green/recreational area are two strong predictors of place attachment, developers and local governments alike should take into consideration environmental issues from designing and building to marketing. For example, local governments may want to use incentives to encourage developers to create green spaces within the community.

### Limitations and future research

The study limitations and directions for future research are discussed as follows. First, this study examined a comprehensive set of residential environment elements and its effect on place attachment. Future studies may want to study some psychological variables such as attitude toward eco-friendly housing and personal values. Although our study revealed some differences between males and females, the study could not pinpoint the underlying causes. Future studies may want to investigate what causes males and females to respond differently toward eco-friendly housing. For example, what makes males more interested in public facilities and eco-friendly material than females? Second, the *R*^2^ value (0.330) for pro-environmental behavior was relatively lower than other *R*^2^ values (0.589–0.650). Future studies may want to consider some other variables (e.g., place environmental concern, Lee et al., 2014a house types, Azimi and Esmaeilzadeh, 2017; comparison of general purchase households and resettled cooperative households, Oh and Lee, 2003) that may account for pro-environmental behavior. This will be a critical issue from the government who wants to promote citizens’ involvement in pro-environmental behavior. Lastly, this study was conducted in Seoul, S. Korea. Seoul is a city full of condominiums with little green space. The importance rating of residential environment elements may be different for other areas (e.g., suburban area, rural area). Future studies may want to collect data from different residential areas to compare the results.

## Data availability statement

The raw data supporting the conclusions of this article will be made available by the authors, without undue reservation.

## Author contributions

JS, J-JY, and Y-KL designed the study, collected the data, and contributed to manuscript writing, and data analysis. SC contributed to the literature review, manuscript writing, and data analysis. All authors contributed to the article and approved the submitted version.

## Funding

This research was supported by the Sejong University Graduate School Specialization Fund in 2022.

## Conflict of interest

The authors declare that the research was conducted in the absence of any commercial or financial relationships that could be construed as a potential conflict of interest.

## Publisher’s note

All claims expressed in this article are solely those of the authors and do not necessarily represent those of their affiliated organizations, or those of the publisher, the editors and the reviewers. Any product that may be evaluated in this article, or claim that may be made by its manufacturer, is not guaranteed or endorsed by the publisher.
